# Supplementary material for: Optimization and Development of Selective Histone Deacetylase Inhibitor (MPT0B291)-Loaded Albumin Nanoparticles for Anticancer Therapy
Source: Pharmaceutics. 2021 Oct 19;13(10):1728. doi: 10.3390/pharmaceutics13101728 (PMC8541575; doi:10.3390/pharmaceutics13101728)
Supplement: Supplementary file 1 [file pharmaceutics-13-01728-s001.zip › pharmaceutics-1398193 - supplementary.pdf]

pharmaceutics

Supplementary Materials: Optimization and Development of Selective Histone Deacetylase Inhibitor (MPT0B291)-Loaded Albumin Nanoparticles for Anticancer Therapy

Athika Darumas Putri, Pai-Shan Chen, Yu-Lin Su, Jia-Pei Lin, Jing-Ping Liou and Chien-Ming Hsieh

Table 1. The 50% inhibitory concentration (IC50) of free MPT0B291 and MPT0B291-human serum albumin (HSA) nanoparticles (NPs).

Group	IC_{50} (μM)
Free MPT0B291	4.71
MPT0B291-HSA NPs	4.28
